# Precision Immunotherapy for Sepsis

**DOI:** 10.3389/fimmu.2018.01926

**Published:** 2018-09-05

**Authors:** Annemieke M. Peters van Ton, Matthijs Kox, Wilson F. Abdo, Peter Pickkers

**Affiliations:** ^1^Department of Intensive Care Medicine, Radboud University Medical Center, Nijmegen, Netherlands; ^2^Radboud Center for Infectious Diseases, Radboud University Medical Center, Nijmegen, Netherlands

**Keywords:** sepsis, hyperinflammation, immunoparalysis, immunosuppressive therapy, immunostimulatory therapy, biomarkers, precision medicine

## Abstract

Decades of sepsis research into a specific immune system-targeting adjunctive therapy have not resulted in the discovery of an effective compound. Apart from antibiotics, source control, resuscitation and organ support, not a single adjunctive treatment is used in current clinical practice. The inability to determine the prevailing immunological phenotype of patients and the related large heterogeneity of study populations are regarded by many as the most important factors behind the disappointing results of past clinical trials. While the therapeutic focus has long been on immunosuppressive strategies, increased appreciation of the importance of sepsis-induced immunoparalysis in causing morbidity and mortality in sepsis patients has resulted in a paradigm shift in the sepsis research field towards strategies aimed at enhancing the immune response. However, similar to immunosuppressive therapies, precision medicine is imperative for future trials with immunostimulatory compounds to succeed. As such, identifying those patients with a severely suppressed or hyperactive immune system who will most likely benefit from either immunostimulatory or immunosuppressive therapy, and accurate monitoring of both the immune and treatment response is crucial. This review provides an overview of the challenges lying ahead on the path towards precision immunotherapy for patients suffering from sepsis.

## Introduction

Sepsis is defined as life-threatening organ dysfunction caused by a dysregulated host response to infection (1). It is the number one cause of death in the Intensive Care unit (ICU), and the worldwide incidence of sepsis is estimated to exceed 30 million cases per year (2, 3). Despite advances in ICU management and goal-directed interventions in the last decades, sepsis mortality rates remain as high as 30% (4). In the Western world alone, annually an estimated 6 million people die of sepsis, representing more deaths than lung, breast and colon cancer together (5). In addition, it is also one of the most expensive conditions encountered in hospitals, with annual costs exceeding 20 billion dollars in the US alone^1^. Despite these alarming facts, sepsis remains a relatively neglected condition that is unknown to the general public.

In May 2017, the WHO adopted a resolution aimed to improve the prevention, diagnosis, and management of sepsis^2^, illustrating the urgency of the problem. The search for a specific immune system-targeting adjunctive therapy has dominated the sepsis research field for more than 4 decades, with the disappointing result of dozens of negative trials and not a single adjunctive treatment in current clinical use. Experts agree that this is not due to the fact that the drugs tested are ineffective *per se*, but rather to the inability to restrict treatment to selected patient groups that may actually benefit from a specific type of therapy (6–9). Not surprisingly, it is therefore also agreed upon that precision medicine is imperative for future trials to succeed, but accurate and reliable immunomonitoring is currently not a reality (6, 8–10). For new therapies, it is paramount that we do not make the same mistake that was previously made for immunosuppressive treatments by advocating the use of compounds in all sepsis patients. Instead, we should only target patients who are truly hyperinflamed with immune suppressive drugs and immunoparalyzed patients with immunostimulatory compounds to avoid unnecessary risks of side effects in patient groups with a lower chance of a therapeutic effect and increase the chances of success in patient groups with a higher likelihood of benefit. In addition to immunomodulatory therapy, advocating personalized medicine is also relevant for other promising sepsis treatments, for instance those targeting the well-described metabolic dysfunction observed in these patients. However this is beyond the scope of this review. This review discusses whether we are targeting the right pathophysiological immunological mechanisms and emphasizes the challenges that lie ahead on the path towards precision immunotherapy for septic patients.

### Hyperinflammation in sepsis

**Figure 1:**
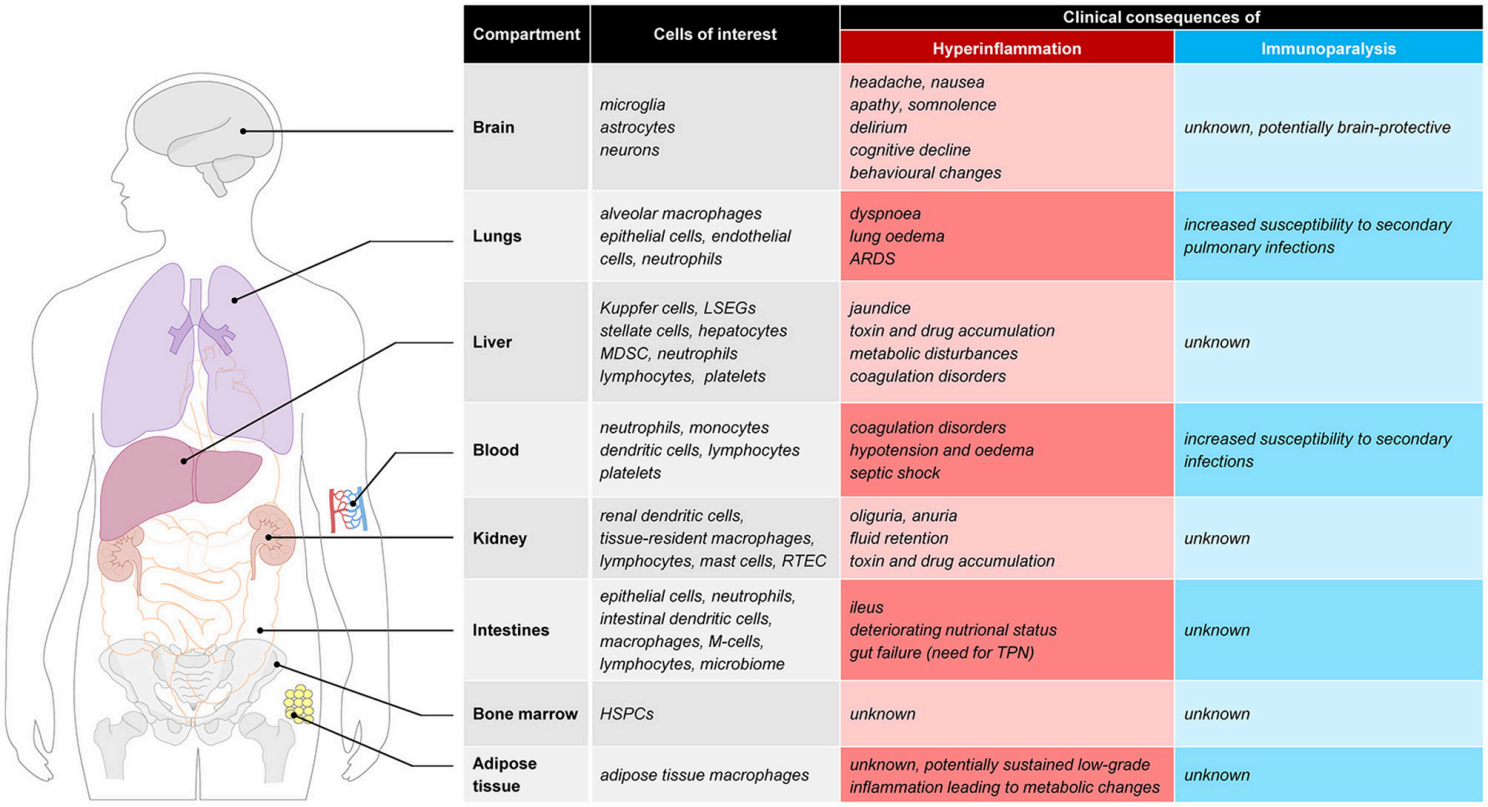
Clinical consequences of hyperinflammation and immunoparalysis to selected tissues. ARDS, acute respiratory distress syndrome; LSEC, liver sinusoidal endothelial cells; MDSC, myeloid-derived suppressor cells; RTEC, renal tubule epithelial cell; M-cell, microfold cell; TPN, total parenteral nutrition; HSPC, hematopoietic stem and progenitor cells.

The proinflammatory response in sepsis is directed at eliminating invading pathogens and involves leukocyte activation, cytokine production, reactive oxygen species and protease release, and complement and coagulation activation (9). An overzealous hyperactive proinflammatory response may exert detrimental effects for the host by eliciting high fevers, hypotension, tachycardia, tachypnoea, coagulation disorders and organ failure, the latter resulting from collateral tissue damage. Examples of deleterious effects of hyperinflammation in various tissues are illustrated in Figure 1. Pulmonary hyperinflammation may lead to the development of acute respiratory distress syndrome (ARDS), a life-threatening condition characterized by unexplained respiratory failure through hypoxemia with bilateral infiltrates of noncardiac origin (11). In the circulating blood compartment hyperinflammation alters coagulation, which may result in a relatively uncommon but fulminant phenomenon called disseminated intravascular coagulation (DIC) (12), which is characterized by simultaneous widespread microvascular thrombosis and profuse bleeding. Capillary leak and development of interstitial oedema are unfortunately very common problems resulting from a systemic hyperinflammatory state, and have widespread cardiovascular effects such as hypotension, tachycardia and in severe cases even septic shock which results in more tissue damage. In the acute phase, hyperinflammation in the brain may cause headaches, nausea, apathy, somnolence or delirium. Furthermore it is suggested that long-term effects such as cognitive decline and behavioral changes can also be attributed to a hyperinflammatory state (13). Other organs that are often affected by hyperinflammation include the kidneys (acute kidney injury which frequently requires renal replacement therapy), the intestines (paralytic ileus), and the liver (liver failure with liver test abnormalities and altered glycemic control).

From the 1970s until the turn of the century, it was commonly assumed that sepsis mortality resulted exclusively from this overzealous pro-inflammatory response. As a consequence, therapeutic research in the sepsis field was solely focused on dampening or preventing excessive inflammation. However, all clinical trials to date investigating immunosuppressive therapy in sepsis, including anti-endotoxin (signaling) molecules, TLR-receptor antagonists, anti-cytokine therapies (e.g., anti-TNF-α, IL-1RA etc.), and high dose corticosteroids(14–24), have convincingly demonstrated that inhibition of the immune response exerts no beneficial effects in an unselected heterogeneous group of sepsis patients.

### Paradigm shift in the understanding of sepsis: the detrimental role of sepsis-induced immunoparalysis

In the last decade a paradigm shift in our understanding of the immune derangements in sepsis has taken place (25). It is increasingly recognized that, for many patients not excessive immune activation, but rather immunosuppression, also known as “sepsis-induced immunoparalysis,” is the overriding immune dysfunction associated with high mortality and morbidity (7, 25–29). The capacity of circulating leukocytes to release proinflammatory cytokines is impaired during immunoparalysis and apoptotic immune cell death is profoundly increased. In contrast to necrotic cell death, which generally causes stimulation of the immune system and enhanced defense against microbes, apoptotic cell death results in anti-inflammatory cytokine production, and cellular anergy (30–32). The majority of cells lost through apoptosis in septic patients are lymphocytes (33) and low absolute lymphocyte counts were shown to be associated with mortality (34). Sepsis-induced immunoparalysis renders patients unable to clear their primary infection and more likely to develop secondary infections with opportunistic bacteria or fungi (35) later on. This is in line with data that the vast majority of septic patients do not die from the initial pro-inflammatory hit, but at a later time point from secondary or opportunistic infections in an immunosuppressed state (35–38). More precisely: approximately a quarter of sepsis patients die within 4 days. Of the remaining three-quarter that survives, one third regains immunocompetence and the mortality in this group is 10%. Two-thirds develops immunoparalysis accounting for 65% of total mortality (10). The development of immunoparalysis appears to start simultaneously with the proinflammatory response. The clinical relevance of immunoparalysis is further illustrated by several observational findings: (i) in patients who died from sepsis or septic shock, a continuous septic focus was observed in 63 of the 71 patients (89%) who were treated with antibiotics for more than 7 days (39), (ii) during the late phase of sepsis, infections due to opportunistic bacteria increase from 9 to 18% and Candida-infections from 13 to 30% (35), and (iii) reactivation of latent viruses was found in 43% of critically ill patients (40). Of interest, detection rate of positive viral PCR results increased with ICU length of stay and was associated with the development of fungal and opportunistic bacterial infections. Importantly, Epstein Barr virus and CMV PCR-titers in patients with a bacterial sepsis was similar to those reported in stem-cell and organ transplant patients, indicative of clinically relevant immune suppression. These recent observational insights indicate that sepsis-induced immunoparalysis accounts for the majority of sepsis-related deaths. Although the importance of immunoparalysis is increasingly recognized (28), there is still lack of consensus that immunosuppression is a clinically important phenomenon (41, 42). Nevertheless, combined with the many disappointing trials on immunosuppressive strategies, appreciation of the detrimental role of sepsis-induced immunoparalysis (7, 25–29, 43) has led the sepsis research field to focus more and more on ways to restore the suppressed immune response through immunostimulatory treatment (7, 25, 28, 29). This concept is appealing and the treatments under investigation are most likely effective to reverse immunoparalysis. However, in light of previous sepsis trials, only patients with proven immunoparalysis should receive immunostimulatory treatment to avoid unnecessary risks and increase the chances of successful trials. However, methods to identify patients with immunoparalysis and subsequent therapeutic reversal of immunoparalysis are still in their infancy.

### Biomarkers to stratify the immune status in sepsis patients

Many cytokines, chemokines or other proteins have been studied as potential biomarkers to characterize a hyperinflammatory state in sepsis patients. Three pro-inflammatory cytokines, namely tumor necrosis factor (TNF), interleukin-1β (IL-1β) and interleukin-6 (IL-6) play a pivotal role in the initial response of the innate immune system to injury or infection. These three cytokines are, among others, crucial for activation of endothelial cells, recruitment of leukocytes to the site, generation of fever and other systemic symptoms, production of acute phase reactants and induction of a shift in cell production in the bone marrow (44). Nevertheless neither TNF or IL-1β have emerged as reliable biomarker for hyperinflammation in sepsis patients, potentially because they are elevated only for a very short period of time in the initial phase of sepsis, when patients may not yet have been admitted to the ICU. IL-6 has been most extensively studied as a potential biomarker, with the advantages of being elevated for a longer period of time and the availability of commercial bedside immunoassays (45). Elevated levels of IL-6 in septic patients have been shown to be associated with increased mortality (46, 47). However this illustrates prognostic rather than diagnostic value and like most cytokines, IL-6 is not specific for sepsis as increased levels are observed in many inflammatory conditions. Moreover the chemokines interleukin-8 (IL-8) and monocyte chemoattractant protein-1 (MCP-1) have been shown to be superior to IL-6 for diagnosis of sepsis (48) and prediction of sepsis mortality (49) respectively. The acute phase protein CRP has a high sensitivity for detection of early onset sepsis (50), but its low specificity is a major drawback for its use as a biomarker to stratify the immune status in patients with sepsis. Procalcitonin (PCT) is elevated in patients with invasive bacterial infections (51). However it remains to be determined whether the detection of bacteraemia with PCT can accurately distinguish patients in a hyperinflammatiory state from patients with immunoparalysis. Research on monocyte activation markers as potential biomarkers of a hyperinflammatory state in sepsis has identified a possible role for the soluble form of the receptor for advanced glycation end-products (sRAGE). This molecule may be considered as a receptor for danger associated molecule patterns (DAMPs) and elevated levels were shown to be associated with poor survival in severe sepsis (52). Despite the numerous laboratory options illustrated above no accurate single biomarker for hyperinflammation in sepsis is currently used in clinical practice. As an alternative, a more clear clinical example for a severe hyperinflammatory state is the macrophage activation syndrome (MAS). This is defined as a fulminant cytokine storm concurrent with hepatosplenomegaly, liver dysfunction, hyperferritaemia, pancytopenia, and disseminated intravascular coagulation (53). MAS is a serious complication of sepsis and the clearly overriding hyperinflammatory state in these patients, may provide opportunities for targeted application of anti-inflammatory therapies, as discussed elsewhere in this review.

Identification of patients with immunoparalysis is currently based on HLA-DR expression on circulating monocytes and, to a lesser degree, cytokine production of leukocytes stimulated *ex vivo* with lipopolysaccharide (LPS, endotoxin) (54–57), although the accuracy of these markers still lacks solid evidence. There are no data on the predictive value on the individual patient level, meaning that precision immunostimulatory treatment for sepsis patients may not be feasible using the markers that are currently advocated. The inability to restrict immunostimulatory treatment to those patients who will actually most likely benefit from it may result in another series of failed clinical trials, because beneficial effects in immunoparalyzed patients will be offset by possible harmful effects of these compounds in immunocompetent patients. Several other markers of immunoparalysis are proposed (25), based on the increasing knowledge of the pathophysiology of immunoparalysis. These include expression of inhibitory receptors like programmed death-1 (PD-1) and its ligand PD-L1 (58), cytotoxic T lymphocyte antigen-4 (CTLA-4) (59), and B and T lymphocyte attenuator (BTLA) (60), molecules that play a role in the exhaustion of lymphocytes. Furthermore, several immunosuppressive lymphocyte subpopulations (including T-regulatory cells) have been identified in patients suffering from immunoparalysis (61). Moreover, epigenetic changes were shown to be involved in immunoparalysis (54), revealing that TLR-induced chromatin modifications are responsible for transient silencing of tolerizable (T) genes (including those encoding proinflammatory mediators), and for priming of non-tolerizable (NT) genes (including those encoding antimicrobial peptides). The T genes are transiently inactivated to prevent pathology associated with excessive inflammation, while the NT genes remain inducible to provide continuous protection from infection and tissue repair. In addition, it was demonstrated that negative TLR regulators such as IRAK-M and SHIP-1, might also participate in the development of immunoparalysis (62). However, the role of these relatively recently discovered mechanisms in the pathogenesis of immunoparalysis is currently unclear and clinical application as a biomarker is not yet feasible.

Understanding what causes the contrasting hyperinflammatory and immunoparalyzed phenotypes observed in sepsis—in other words: why do some patients exhibitit a prevailing hyperinflammatory response while others display immunoparalysis—would greatly aid biomarker discovery and development. To the best of our knowledge, this is currently unknown and a multifactorial etiology is likely, including host-related factors such as age, gender, comorbidities, (epi)genetic predisposition, microbiome composition, expression levels of pattern-recognition receptors (PRRs), and release of DAMPs as well as pathogen-related factors such as the type of pathogen, its virulence and load, and quorum sensing.

Whether it concerns hyperinflammation or immunoparalysis, it appears implausible that a single marker can act as a reliable tool to guide immunomodulating therapy since biomarkers are often related to one or a limited number of pathophysiological mechanisms/pathways, while it has become clear that multiple pathways are activated or inhibited at the same time in sepsis. Therefore, it appears likely that a panel of markers reflects the immune status of the sepsis patient more accurately.

### Compartmentalization of the immune response

At this moment, plasma markers or expression of molecules in/on circulating immune cells are used to identify the immune status of sepsis patients. However, due to compartmentalization of the immune response and temporal differences in the immune response between compartments, the phenotype of blood leukocytes may not always be reflective of the current immune status. There are several observations in support of this notion. For instance, leukopenia is associated with a more pronounced cytokine response in animal models of sepsis (63), indicating limited importance of blood immune cells in producing inflammatory mediators that are important for host defense. Furthermore, the compartmentalized nature of the immune response is supported by several results obtained by our group in the human endotoxemia model. In this model endotoxin [also known as lipopolysaccharide (LPS)] is administered to healthy volunteers. Numerous studies have established that the immune response to LPS captures many hallmarks of the immune response observed in sepsis, including a phase of immune suppression. The latter phenomenon is known as “endotoxin tolerance,” and characterized by a blunted inflammatory response upon subsequent LPS challenges *in vivo* and *ex vivo* (64). In keeping with the overlap between LPS-elicited effects and clinical sepsis, endotoxin tolerance bears many similarities to sepsis-induced immunoparalysis, including decreased cytokine production by circulating leukocytes and attenuated mHLA-DR expression (25, 27, 43). Therefore, endotoxin tolerance has been used in translational research to model and investigate treatments for sepsis-induced immunoparalysis, both *in vitro* (65) and *in vivo* in humans (43). Strikingly, however, animal studies have shown that despite a diminished immune response indicating endotoxin tolerance, pathogen clearance and survival upon a live bacterial challenge were improved in mice pre-treated with LPS or other TLR ligands (66–69). It is unknown whether these counterintuitive findings of an enhanced defense against pathogens concurrent with endotoxin tolerance are also present in humans, which should be an important focus for future studies. Nevertheless, using the human endotoxemia model we have demonstrated profound differences in *ex vivo* and *in vivo* endotoxin tolerance kinetics. Both mHLA-DR expression (43) and *ex-vivo* cytokine production by stimulated leukocytes (70–72) are suppressed rapidly in the first hours after endotoxin administration. The same studies show that these markers normalize quickly afterwards and are restored within 24 h after endotoxin administration, indicating that functionality of circulating leukocytes can quickly recover in the absence of ongoing inflammation. In contrast, the *in vivo* response 1 to 2 weeks after the first endotoxin administration is still severely blunted with an attenuation of pro-inflammatory cytokine levels of approximately 60% compared to the first endotoxin challenge (43, 70). From these observations it could be concluded that parameters measured in the blood compartment may not reflect the responsiveness of the immune system as a whole, and that immune cells in other compartments than the blood may likely better reflect the *in vivo* immune status at a given moment in time. It is currently unknown whether this discrepancy between the immune status of cells within the blood compartment and the responsiveness of the *in vivo* immune system is of clinical importance in sepsis patients, but tissue resident macrophages and not circulating immune cells appear to be predominantly responsible for the innate immune response in sepsis. The relevance of organ-specific immunology and possible consequences of a hyperinflammatory or immune-suppressed state in several tissues (e.g., lungs, brain, adipose tissue, and bone marrow), are graphically presented in Figure 1 and outlined below.

The lungs likely represent a highly relevant compartment in the context of sepsis-induced hyperinflammation and immunoparalysis. An exaggerated pro-inflammatory response in the lungs may result in the life-threatening condition of ARDS, requiring invasive mechanical ventilation and complex respiratory therapy (73). In contrary, an immunosuppressed response as observed in immunoparalysis increases the susceptibility to secondary pulmonary infections. Along these lines, hospital-acquired pneumonia represents the secondary infection with the highest incidence observed in patients that recovered from their initial sepsis (74, 75, 42). Alveolar macrophages (AMs), as the first line of defense in the lungs, play a key role in host defense. Whereas previous work has demonstrated that AMs display a primed rather than tolerant phenotype shortly (1.5–6 h) after LPS administration in healthy volunteers (76), small studies in septic patients point toward a tolerant phenotype of AMs (77, 78). This may indicate that AMs switch from an initially primed phenotype to a tolerant immunosuppressed phenotype at later stages of disease progression, but these dynamics have yet to be unraveled.

The brain was long regarded as an immune privileged organ. The last decades of research have shown an important immunological role of the brain in “non-immunological” diseases like dementia, or psychiatric diseases. The so-called microglial cells represent the resident macrophage population in the brain, and account for 5–20% of the total glial cell population of the brain. Research in healthy humans (79), animal data (80) and post-mortem brain tissue of patients suffering from severe systemic inflammation (81, 82) show that systemic inflammation is a strong trigger that activates the resident microglia (macrophages) of the brain. During systemic inflammation, systemic inflammatory cytokines can enter brain tissue due to a disrupted blood-brain barrier, but also through several parts of the brain that lack a blood-brain barrier and directly activate microglial cells. As a result, systemic inflammation may result in an exaggerated neuroinflammatory cascade, which disrupts normal homeostasis and cell function and may lead to neuronal cell loss and cognitive deterioration (83, 84). Neuroinflammation is thought to contribute to both acute sepsis-associated encephalopathy, as well as long-term cognitive impairment following critical illness. Previously, research into immune responses of the brain during systemic inflammation was impossible in living patients as the brain is a body compartment not accessible for immunological research purposes without using the invasive procedure of brain biopsies. Recently, several innovative nuclear imaging tracers have been developed that can quantitatively measure microglial activation *in vivo*, by targeting the mitochondrial 18 kDa translocator protein (TSPO). Systemic inflammation evoked during experimental human endotoxemia was demonstrated to induce a 30–60% increase in microglial activation in healthy volunteers 3 h after administration of endotoxin (79). However, patients suffering from sepsis often develop more prolonged periods of systemic inflammation and a subsequent immunosuppressive state. The longitudinal effects of endotoxemia and systemic inflammation on tissue resident macrophage activation in the brain are unclear. A recent study in mice showed that repeated subjection to systemic inflammation on consecutive days induced a brain-specific training effect initially, followed by reduced immunological response (immune tolerance) of the brain after successive stimuli (85). In addition, a recent human study in prostate surgery patients found decreased microglial activity, measured by reduced microglial nuclear ligand binding, 3–4 days postoperatively compared to baseline (86). These results are the first indications that innate immunity responses in the brain show signs of immunoparalysis, coinciding with *ex vivo* and *in vivo* peripheral immunoparalysis.

Recent studies have demonstrated that microbial components can directly interact with hematopoietic stem and progenitor cells (HSPCs) in the bone marrow via Toll-like receptors (TLRs) expressed on HSPCs (87, 88). Microbial sensing by HSPCs during infection may therefore influence hematopoietic cell division and differentiation (88), and may ultimately impact the efficacy of host defenses during infection. Moreover, septic patients also exhibit reduced expression of HLA-DR in the myeloid lineage of the bone marrow (89). To date, the effects of systemic inflammation on HSPCs and their role in development and maintenance of hyperinflammatory responses or immunoparalysis in humans are unknown and represents an exciting field for further study.

Adipose tissue is increasingly recognized as an immunological organ, containing substantial amounts of adipose tissue macrophages (ATMs) (90). Advances on the interplay between metabolic and immunological research suggest an important role of the immune system in metabolic conditions such as obesity and diabetes mellitus (91). Lipids are important signaling moieties for both immune responses and metabolic regulation. Lipid infusion *in vivo* activates TLR4 signaling in adipocytes and macrophages and enhances inflammatory gene expression in adipose tissue (92) which adds to systemic insulin resistance. During obesity, many immune cells infiltrate in adipose tissue and promote a low-grade chronic inflammation (91). Conversely is the role of adipose tissue during systemic inflammation (e.g., sepsis) less well studied and human studies concerning the dynamics of immune suppression/tolerance in ATMs are completely lacking.

Future studies to characterize the immune response in other body compartments are ongoing and necessary to further characterize the *in vivo* immune status. These studies will improve the insight in the mechanistics of the immunological response during sepsis. It needs to be acknowledged that guiding immunotherapy in clinical practice based on other compartments than blood will however not be readily applicable, since tissue-resident immunological markers are not easily harvested or measured.

### Novel treatment modalities in sepsis

As mentioned earlier, previous therapeutic strategies for sepsis patients have virtually exclusively focused on blocking inflammation early in the course of sepsis. It has become clear that a considerable proportion of sepsis patients do not die from an overwhelming immune response and that suppressing the immune system is not an effective strategy when applied to all sepsis patients. Nevertheless, it is conceivable that a subgroup of hyperinflamed patients may have benefited from immunosuppressive therapy if they had been treated according to their immune status. For example, the phase 3 trial evaluating the immune suppressant IL-1 receptor antagonist (anakinra), performed more than 20 years ago, revealed no effect on mortality in severe sepsis patients (21). However, a *post-hoc* analysis of this study published many years later (93) demonstrated a significantly lower mortality in a subgroup of patients with MAS, which represented approximately 6% of the enrolled sepsis patients. Although no definitive conclusions can be drawn from this *post-hoc* analysis, it does suggest that a specific subgroup of patients in a hyperinflammatory state could benefit from immune suppressive therapy. Another argument that therapy aimed at inhibition of the immune response should not be discarded as of yet comes from a meta-analysis of 17 randomized controlled trials (almost 9,000 patients) evaluating the effects of anti-tumor necrosis factor alpha (TNF-α) therapy on mortality in severe sepsis. Despite negative results in each individual study, the pooled odds ratio showed a significantly reduced 28-day all cause mortality (94).

In the last decade, several immunostimulatory treatments have shown promise in preclinical, as well as in case series and/or small clinical studies (58, 95–104). Granulocyte-macrophage colony stimulating factor (GM-CSF) and interferon-gamma (IFNγ) are the most extensively investigated immunostimulatory agents in sepsis. These compounds are potent stimulators of myeloid cell function and they potentiate antigen presentation capabilities through increasing mHLA-DR expression on and pro-inflammatory cytokine production by monocytes (105, 106). A biomarker-guided (inclusion criterion mHLA-DR>8000 monoclonal antibodies per cell) randomized controlled trial comparing GM-CSF to placebo showed that GM-CSF was safe and effective in restoring monocytic immunocompetence. Exploratory endpoints suggested that treated patients had shorter duration of mechanical ventilation and a more swift decrease of disease severity scores (95). Although a meta-analysis of 4 RCT's did not reveal a beneficial effect of GM-CSF on 28-day mortality, patient numbers were probably too small for evaluation of this endpoint (107). Treatment with IFNγ resulted in increased mHLA-DR expression and restored TNFα production in a human endotoxemia study, while further attenuating production of the key anti-inflammatory cytokine IL-10 (43). Furthermore, IFNγ showed promising results in several small case series, for instance in patients suffering from opportunistic infections not responding to regular treatment (97). Targeting lymphocyte loss with apoptosis inhibitors has shown potential in animal studies (108–110) but could theoretically result in uncontrolled cell growth and organ injury as consequence of neutrophil accumulation in tissues. Recently, the IRIS-7 randomized controlled phase 2 trial was published, in which 27 patients with septic shock were treated with recombinant human IL-7 or placebo (111). In this trial, severe lymphopenia was used to identify immunosuppressed patients. The anti-apoptotic and lymphocyte function-enhancing cytokine IL-7 was well tolerated and reversed sepsis-induced lymphopenia in these patients. Naturally, statistical power to demonstrate clinically relevant treatment effects was inadequate. Another attractive immunostimulatory target is blockade of programmed death-1 (PD-1) or its ligand PD-L1. The PD-1 system is upregulated in sepsis patients (37) and inhibition of the interaction between PD-1 and its ligands promotes immune responses and antigen-specific T-cell responses. In recent years, positive responses to anti-PD-L1 therapy was demonstrated in the field of oncology and given the many similarities between the immunosuppressive mechanisms in cancer and sepsis, this could be promising for the potential of PD-L1 antagonism in sepsis-induced immunoparalysis. Unfortunately, a large multicenter trial was recently aborted by the sponsor due to other priorities (“CA209-9FH, a randomized, double-blind, placebo-controlled, parallel-group study to evaluate the efficacy and safety of nivolumab in adults with sepsis”). Table 1 summarizes the mechanism of action and the (clinical) evidence thus far of the immunomodulatory compounds described in this paragraph.

**Table 1:** Examples of immunotherapy in sepsis.

	**Mechanism of action**	**Summary of evidence**
**IMMUNOSUPPRESSIVE COMPOUNDS**		
anti-TNFα (various)	Blocks pro-inflammatory effects of TNFα	- Individual studies: no beneficial effects (94) - Meta-analysis: reduced 28-day mortality, OR = 0.91 [95% CI 0.83–0.99] (94)
IL-1RA (anakinra)	Blocks IL-1 receptor → inhibits downstream pro-inflammatory effects	- Study in unselected population of severe sepsis patients: no effect on mortality (21) - *Post-hoc* analysis in subgroup of hyperinflamed patients with macrophage activation syndrome: lower mortality (93)
**IMMUNOSTIMULATORY COMPOUNDS**		
GM-CSF	Enhances antigen presenting capacity and pro-inflammatory cytokine production	- Meta-analysis: no effect on 28-day mortality in sepsis patients (probably underpowered) (107) - Biomarker-guided study (based on mHLA-DR expression): restoration of monocytic immunocompetence, shorter duration of mechanical ventilation, and more swift improvement of disease severity scores as exploratory endpoints (95)
IFN-γ	Enhances antigen presenting capacity and pro-inflammatory cytokine production	- Human endotoxemia model (mimicking sepsis-induced immunoparalysis): increased mHLA-DR expression, restored TNFα production and further attenuated IL-10 production (43) - Case series in patients suffering from opportunistic infections not responding to regular treatment: increased mHLA-DR expression and cytokine production by *ex vivo-*stimulated leukocytes (97)
Recombinant human IL-7	Reduces apoptosis and enhances lymphocyte function	- Phase 2 trial in septic shock patients with severe lymphopenia: safe, well-tolerated and reversal of lymphopenia (111)
anti-PD-(L)1	Inhibits PD-1-PD-L1 interaction → reduces apoptosis and promotes T-cell responses	- Preclinical data in sepsis models: promising results (e.g., prevention of sepsis-induced depletion of lymphocytes, increased TNF-α and IL-6 production, decreased IL-10 production, enhanced bacterial clearance, improved survival (102) - Clinical data in the oncology field: effective, especially in advanced melanoma and non-small cell lung cancer. - No clinical trials in sepsis patients yet.

*TNFα, tumor necrosis factor alpha; IL1RA, Interleukin-1 receptor antagonist; IL-1, interleukin-1; GM-CSF, granulocyte-macrophage colony stimulating factor; IFNγ, interferon gamma; IL-7, interleukin-7; anti-PD-L1, programmed death-1 ligand antagonist; OR, odds ratio*.

Fortunately, a number of clinical trials investigating these and other immunostimulatory treatments are currently underway or planned, illustrating the current interest and relevance of this type of treatment for sepsis^3^. However, these either do not use markers to enrich their patient population, or use biomarkers of which the accuracy and robustness has not been sufficiently demonstrated.

### Summary and future directions

This review highlights the current challenges we face toward precision immunotherapy for patients suffering from sepsis. The urgent need for a patient-tailored approach in sepsis treatment is clear, as 40 years of undirected sepsis trials have not resulted in a single adjunctive therapy in current clinical use. Clearly, we need tools to determine whether hyperinflammation or immune suppression is the overriding immune dysfunction in a specific patient. The paradigm shift in the understanding of the pathophysiology of sepsis has resulted in increased interest for promising immunostimulatory therapies, but it is key to identify and select the appropriate patient population who may most likely benefit from these compounds. So far, current circulating biomarkers measured in blood have not been found sufficiently robust for use in clinical practice. From a pathophysiological perspective studies into other body compartments are warranted to increase our understanding of the *in vivo* immune response in patients with sepsis, but these insights will not be easily translated in feasible methodology for clinical practice. As such, finding a reliable biomarker to classify and monitor the overall immune response in patients with sepsis and to guide and personalize immunotherapy remains a holy grail.

## Author contributions

MK and AP drafted, and WA and PP revised the manuscript. All the authors read and approved the final version of the manuscript.

### Conflict of interest statement

The authors declare that the research was conducted in the absence of any commercial or financial relationships that could be construed as a potential conflict of interest.

## Footnotes

^1^*Agency for Healthcare Research and Quality Healthcare Cost and Utilization Project Statistical Brief No 160*. Available online at: https://www.hcup-us.ahrq.gov/reports/statbriefs/sb160.pdf. 2013; August.

^2^Available online at: https://www.global-sepsis-alliance.org/news/2017/5/26/wha-adopts-resolution-on-sepsis. WHA resolution sepsis.

^3^Clinicaltrials.gov registration no's: NCT02797431, NCT02576457, NCT02361528, NCT02867267.
